# Optimizing SARS-CoV-2 Immunoassays for Specificity in Dengue-Co-Endemic Areas

**DOI:** 10.7759/cureus.47683

**Published:** 2023-10-25

**Authors:** Nihad Adnan, Md Ahsanul Haq, Taslima Akter Tisha, Shahad Saif Khandker, Mohd. Raeed Jamiruddin, SM Shafiul Alam Sajal, Salma Akter, Md Firoz Ahmed, Rubhana Raqib, Mohib Ullah Khondoker, Nafisa Azmuda, Mainul Haque

**Affiliations:** 1 Microbiology, Jahangirnagar University, Dhaka, BGD; 2 Bio-Statistics, Infectious Diseases Division, International Centre for Diarrhoeal Disease Research, Bangladesh (icddr, b), Dhaka, BGD; 3 Biochemistry and Molecular Biology, Gonoshasthaya Samaj Vittik Medical College, Dhaka, BGD; 4 Pharmacy, Bangladesh Rural Advancement Committee (BRAC) University, Dhaka, BGD; 5 Immunology, Nutrition, and Toxicology Laboratory, Infectious Diseases Division, International Centre for Diarrhoeal Disease Research, Bangladesh (icddr, b), Dhaka, BGD; 6 Community Medicine, Gonoshasthaya Samaj Vittik Medical College, Dhaka, BGD; 7 Department of Research, School of Dentistry, Karnavati Scientific Research Center (KSRC) Karnavati University, Gandhinagar, IND; 8 Pharmacology and Therapeutics, National Defence University of Malaysia, Kuala Lumpur, MYS

**Keywords:** maximize, precision, dengue co-endemic areas, sensitivity specificity, elisa, immunoassay, spike protein, cross-reactivity, dengue, covid-19

## Abstract

Introduction

The overlap in clinical presentation between COVID-19 and dengue poses challenges for diagnosis in co-endemic regions. Furthermore, there have been reports of antibody cross-reactivity between SARS-CoV-2 and dengue. Our research aims to evaluate SARS-CoV-2 antigens for serological testing while reducing the possibility of cross-reactivity with anti-dengue antibodies.

Method

Two hundred and ten serum samples were collected from 179 patients and divided into four panels. Panels 1 and 2 consisted of COVID-19-negative healthy donors (n=81) and pre-pandemic dengue patients (n=50), respectively. Alternatively, Panel 3 (n=19) was composed of reverse transcription-quantitative polymerase chain reaction (RT-qPCR)-positive samples collected within two weeks of COVID-19 symptom onset, while Panel 4 (n=60) was composed of positive samples collected after two weeks of symptom onset. Previously developed and characterized in-house SARS-CoV-2 spike-1 (S1), receptor binding domain (RBD), and nucleocapsid (N) immunoglobin G (IgG)-enzyme-linked immunosorbent assay (ELISA) assays were used for the study.

Results

Six dengue-positive sera cross-reacted with the RBD of SARS-CoV-2. However, only one dengue-positive sera cross-reacted with the S1 and N proteins of SARS-CoV-2. Co-immobilization of S1 and RBD in different ratios revealed an 80:20 (S1:RBD) ratio as optimal for achieving an overall 96.2% sensitivity with the least cross-reaction to anti-dengue antibodies.

Conclusion

Our findings indicated that SARS-CoV-2 RBD-based immunoassays present more cross-reactivity with anti-dengue antibodies than S1 and N proteins. Furthermore, co-immobilization of S1 and RBD reduces the cross-reactivity with anti-dengue antibodies compared to RBD, thereby increasing the immunoassay specificity without affecting overall sensitivity for the dengue-endemic areas.

## Introduction

The co-endemicity of dengue fever and COVID-19, especially in tropical and subtropical areas of the world, has shaken the healthcare sector once again, which gradually started recovering by introducing COVID-19 vaccines [1-3]. In 2023, more than 3.7 million dengue cases were reported globally, with the number still rising with the emergence of Omicron (BA.2.86), a SARS-CoV-2 variant [4,5]. Unfortunately, despite the rollout of current vaccines, new COVID-19 variants are still emerging [6]. These dual attacks by COVID-19 and dengue generate further economic downfall, especially in the dengue-affected regions.

Severe acute respiratory syndrome coronavirus 2 spreads primarily through droplets from coughing and sneezing [7], whereas dengue virus (DENV) is transmitted through mosquito bites [8]. However, SARS-CoV-2 and DENV belong to different families, i.e., *Coronaviridae *(*Orthocornavirinae *subfamily) and *Flaviviridae*, respectively. Early symptoms coincide, such as high fever, myalgia, nausea, headache, leukopenia, and thrombocytopenia [9,10]. Co-infection and misdiagnosis have been reported since the beginning of the COVID-19 pandemic [10-13]. 

The ubiquity of molecular testing platforms in developing and developed countries ensured early diagnosis of COVID-19, aiding in combating the pandemic. With the evolution of the disease, multiple low-cost, high-sensitivity tests such as antigen tests, reverse transcription-quantitative polymerase chain reaction (RT-qPCR), enzyme-linked immunosorbent assay (ELISA), matrix-assisted laser disruption ionization time-of-flight mass spectroscopy (MALDI-ToF-MS), loop-mediated isothermal amplification (LAMP), and others have evolved [14-17]. On the other hand, dengue fever is primarily diagnosed virologically or serologically, the latter being more widely practiced [18,19].

Severe acute respiratory syndrome coronavirus 2 has 12 putative functional open reading frames (ORF), which translate to non-structural proteins and structural proteins such as nucleocapsid (N), envelope (E), membrane (M), and spike (S) [20]. The trimeric spike protein has two subunits: S1, which comprises the receptor-binding protein, and S2, which forms the fusion protein. Due to its significance in viral entry, diagnostic tools and vaccines targeting the spike protein are being developed [21-24]. On the other hand, the dengue virus has four serotypes, namely DENV 1-4, sharing 65% of genomic similarity [25]. The DENV genome encodes for three structural proteins, namely, envelope (E), capsid (C), and membrane (M), and seven non-structural proteins, namely, NS1, NS2A, NS2B, NS3, NS4A, NS4B, and NS5, with NS1 being evaluated as an early diagnostic marker [26].

Several serodiagnosis assays have been manufactured targeting N, S, or receptor binding domain (RBD) proteins for SARS-CoV-2 [27]. These kits can detect pan-immunoglobin (Ig), IgG, IgM, or IgA following COVID-19 infection [28]. However, several studies have reported co-endemicity and cross-reaction between COVID-19-positive sera with anti-dengue IgG/IgM kits and vice versa [10,11]. Moreover, some dengue NS1 and IgM rapid diagnostic tests (RDTs) are also reported to cross-react with Zika and Chikungunya virus-infected sera [19]. Thus, it is essential to properly evaluate RDTs and other serodiagnosis assays for their cross-reactivity with closely related pathogens with whom the clinical manifestations coincide before being introduced to the general population. This study aims to evaluate pre-COVID-19 pandemic dengue sera for their potential cross-reaction with SARS-CoV-2 S1, RBD, and N proteins using previously developed in-house ELISAs. Moreover, immunoassays were optimized to minimize the cross-reaction without affecting sensitivity.

A portion of this paper was published earlier on the medRxiv preprint server on December 22, 2020.

## Materials and methods

Specimen selection and assortment

**Figure 1:**
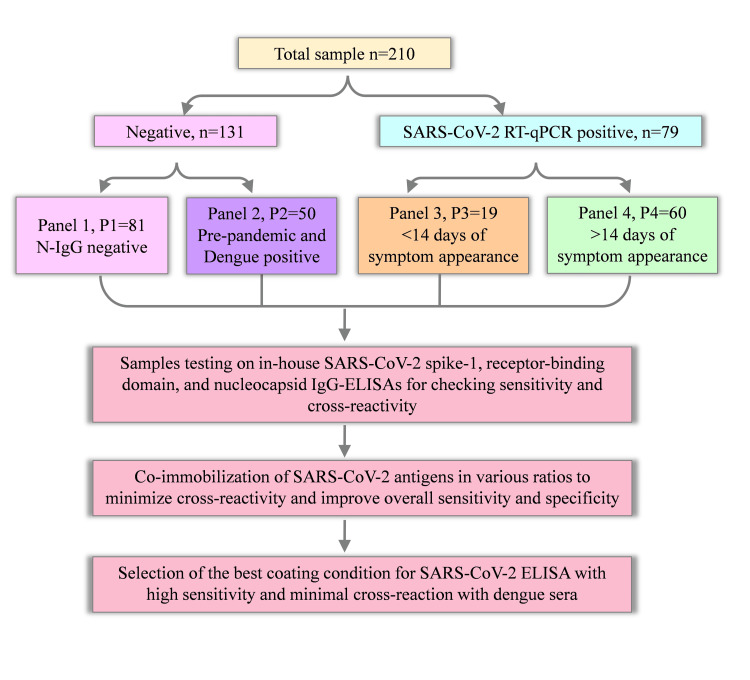
Overview of the study RT-qPCR: reverse transcription-quantitative polymerase chain reaction; N: nucleocapsid; IgG: immunoglobin G; ELISA: enzyme-linked immunosorbent assay

To characterize the serological cross-reaction between SARS-CoV-2 and DENV, serum samples (n=210) were collected from 179 individuals (Figure 1).

The negative sera were categorized into two panels (P): P1 samples collected from individuals with no COVID-19 symptoms. They were N-IgG negative from April to June 2020 (n=81), and P2 samples were collected from pre-pandemic dengue-infected individuals (n=50). Seventy-nine positive serum samples were collected from confirmed RT-qPCR-positive SARS-CoV-2 individuals. These positive samples were categorized into two panels: P3 contained sera from SARS-CoV-2 qRT-PCR positive patients with <14 days of symptom appearance (n=19), and P4 contained sera from SARS-CoV-2 positive patients with >14 days from symptom onset (n=60). All specimens were aliquoted in small volumes and preserved at -80 °C for further analysis.

Stratification of COVID-19 and dengue samples for seropositivity and seronegativity

The samples were characterized based on four factors: clinical symptoms, RT-qPCR test outcomes, the absence of nucleocapsid protein, and samples collected before the pandemic. Following the manufacturer's instructions, seropositive dengue samples (P2) were re-characterized using the dengue IgG/IgM immunoassay Scan® dengue IgG+IgM card test (Bhat BioTech, Bangalore, India). Furthermore, P1, P3, and P4 were also tested with this same assay for anti-dengue antibodies and confirmed as seronegative.

Preparation of in-house S1-IgG, RBD-IgG, and N-IgG ELISA assays and characterization of samples

Assay development was performed in the 96-well microtiter plates (ExtraGene, Taichung City Taiwan) as previously described [29-31]. Briefly, recombinant S1 (Cat. No. 40591-V08H), RBD (Cat. No. 40592-V08H), and N (Cat. No. 40588-V08B) proteins specific to SARS-CoV-2 were purchased from a commercial source (Sino Biologicals, Beijing, China) and used as capturing agents. The presence or absence of anti-SARS-CoV-2 human IgGs in panels P1-P4 was carried out on these S1-IgG, RBD-IgG, and N-IgG ELISA assays (Figure 1). Horseradish peroxidase (HRP)-conjugated goat anti-human IgG (Native Antigen, Kidlington, UK) was used as a secondary antibody, and color development was observed using the substrate tetramethylbenzidine (TMB) (Dojindo Molecular Technologies, Rockville, MD). The results were observed at 450 nm wavelength using a microplate reader (Thermo-Fisher Scientific, Waltham, MA).

Optimization of immunoassay by co-immobilization of SARS-CoV-2 antigens in different ratios to improve sensitivity and specificity

The SARS-CoV-2 S1 and RBD proteins were immobilized in 100:0, 80:20, 60:40, and 0:100 ratios on the coating surface. After blocking the coated surface, randomly selected cross-reacting dengue sera and non-reactive sera (n=4) were tested on these surfaces. The method mentioned in the previous section was followed for characterization. Panels 1-4 were re-tested in the best condition obtained by co-immobilization and further tested for assay validation (Figure 1).

Ethical statement

The ethical approval to conduct this study was obtained from the National Research Ethics Committee (NREC), the national research ethics body of the Government of Bangladesh. The approval number is BMRC/NREC/2019-2022/697. Informed consent was obtained from all the participants. The study's objectives and information regarding the use of data were provided to participants before sample collection. Clarification was provided to the participants upon their inquiries.

Statistical analysis

Statistical analysis was performed with STATA 15 (StataCorp LLC, College Station, TX) and the graphical representation using Prism version 7.05 (GraphPad Software, La Jolla, CA). A p-value of <0.05 was considered significant. The precision of the in-house ELISA assay compared to that of the true positives was characterized by a 95% confidence interval in its sensitivity, specificity, receiver operating characteristic (ROC), positive predictive value (PPV), and negative predictive value (NPV). Test agreement estimation was carried out using Cohen's Kappa test. We used a univariate regression model to evaluate the mean difference of S1, RBD, and N-IgG titers among P1, P2, P3, and P4 panels. A non-parametric Wilcoxon signed-ranks test was used to see the difference of S1, RBD, and N-IgG titers separately in the overall population: <14 days (P3) and >14 days (P4).

## Results

Characterization of COVID-19 sera using in-house ELISAs

Among the P3 samples, S1-IgG ELISA successfully detected 68.4% (95% CI=43.4%, 87.4%) of cases with a test agreement of 80.4% (kappa=0.762) (Table 1).

**Table 1:** Sensitivity, specificity, and accuracy for S1, RBD, and N antigen against IgG in RT-qPCR positive against SARS-CoV-2 and negative controls S1: spike-1; RBD: receptor binding domain; N: nucleocapsid; CI: confidence interval; PPV: positive predictive value; NPV: negative predictive value; ROC: receiver operating characteristic; RT-qPCR: reverse transcription-quantitative polymerase chain reaction Kohen's kappa test was used to estimate the observed proportion of the test agreement.

Protein	Days	Observation	Sensitivity (95% CI)	Specificity (95% CI)	ROC (95% CI)	PPV (95% CI)	NPV (95% CI)	Kappa	Test agreement	Accuracy
S1	≤ 14 (P3)	n1=19 n2=131	68.4(43.4, 87.4)	99.2(95.8, 100)	0.84(0.73, 0.95)	92.9(66.1, 99.8)	95.6(90.6, 98.4)	0.762	80.4%	95.3%
	>14 (P4)	n1=60 n2=131	100(94.0, 100)		1.0(0.99, 1.00)	98.4(91.2, 100)	100(97.2, 100)	0.988	99.5%	99.5%
	Overall	n1=79 n2=131	92.4(84.2, 97.2)		0.96(0.93, 0.99)	98.6(92.7, 100)	95.6(90.6, 98.4)	0.928	96.7%	96.7%
RBD	≤ 14 (P3)	n1=19 n2=131	84.2(60.4, 96.6)	95.4(90.3, 98.3)	0.90(0.81, 0.98)	72.7(49.8, 89.3)	97.7(93.3, 99.5)	0.746	76.4%	94.0%
	>14 (P4)	n1=60 n2=131	100(94.0, 100)		0.98(0.96, 1.00)	90.9(81.3, 96.6)	100(97.1, 96.6)	0.929	96.9%	96.9%
	Overall	n1=79 n2=131	96.2(89.3, 99.2)		0.96(0.93, 0.99)	92.7(84.8, 97.3)	97.7(93.3, 99.5)	0.909	95.7%	95.7%
N	≤ 14 (P3)	n1=19 n2=131	84.2(60.4, 96.6)	98.4(94.2, 99.8)	0.91(0.83, 1.00)	88.9(65.3, 98.6)	97.6(93.0, 99.5)	0.845	86.5%	96.7%
	>14 (P4)	n1=60 n2=131	98.3(91.1, 100)		0.98(0.96, 1.0)	96.7(88.7, 99.6)	99.2(95.5, 100)	0.963	98.4%	99.0%
	Overall	n1=79 n2=131	94.9(87.5, 98.6)		0.97(0.94, 0.99)	97.4(90.9, 99.7)	96.8(91.9, 99.1)	0.937	97.0%	97.1%

**Figure 2:**
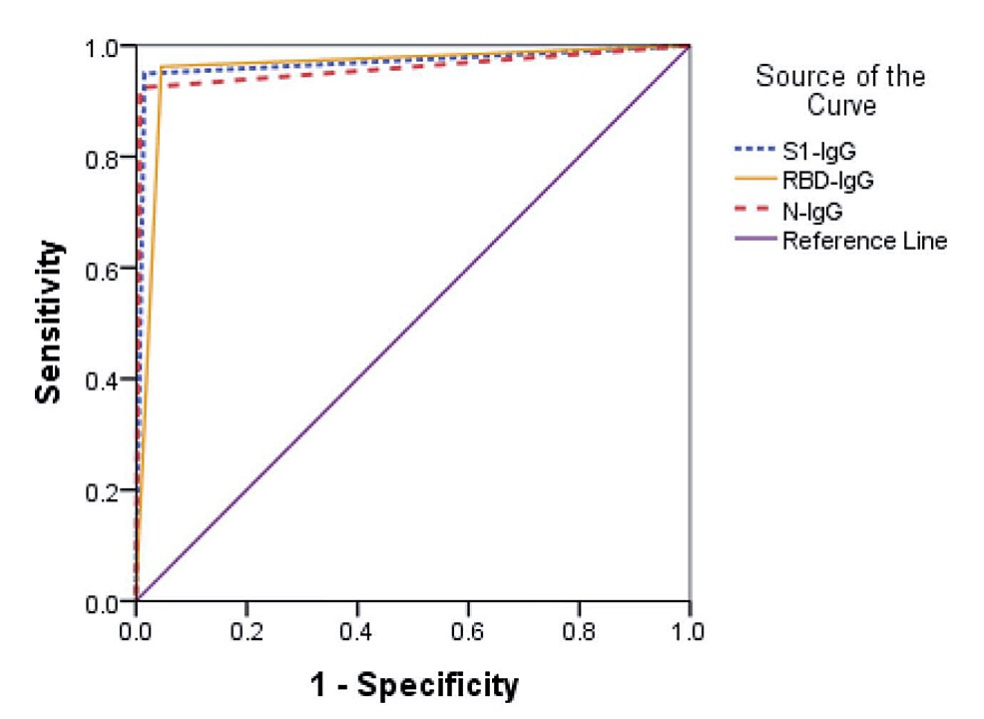
Detection of SARS-CoV-2 S1-IgG, RBD-IgG, and N-IgG among the overall participants; the area under the ROC curve was considered both true and test cases S1: spike-1; IgG: immunoglobin G; RBD: receptor binding domain; N: nucleocapsid; ROC: receiver operating characteristic

The test kit presented NPV and PPV values of 95.6% and 92.9%, respectively. In the P4 samples, the sensitivity was 100% (95% CI = 94.0%, 100%) with a test agreement of 99.5% (kappa=0.988). However, the overall sensitivity and specificity were 92.4% (95% CI: 84.2%, 97.2%) and 99.2% (95% CI: 95.8%, 100%), respectively. It also has a test agreement of 96.7%, and test accuracy was 96.7% (Table 1). Additionally, the overall ROC, PPV, and NPV for S1-ELISA were 0.96 (95% CI=0.95, 0.99), 98.6%, and 95.6%, respectively (Figure 2 and Table 1).

In RBD-specific IgG ELISA, P3 samples presented a sensitivity of 84.2% (95% CI = 60.4%, 96.6%), while P4 samples showed 100% (95% CI=94%, 100%) sensitivity. Both the sample sets presented test agreement of 76.4% (kappa=0.746) and 96.9% (kappa=0.929), respectively, with an overall sensitivity of 96.2% (95% CI= 89.3%, 99.2%) (Table 1). Though the sensitivity was higher than S1-ELISA, the specificity was reduced to 95.4% (95% CI= 90.3%, 98.3%), while kappa test agreement was 95.7% (kappa=0.909) with an overall assay ROC of 0.96 (95% CI= 0.93, 0.99) (Figure 2), PPV of 92.7%, and NPV of 97.7% (Table 1).

The overall sensitivity of N-IgG ELISA was comparable with RBD but slightly better than S1 (Table 1). Like RBD, N also presented better sensitivity of 84.2% (95% CI: 60.4%, 99.2%) during the early phase (P3 samples) with test agreement of 86.5% (kappa=0.845), while sensitivity was slightly lower at 98.3% (95% CI: 91.1%, 100%) compared with RBD and S1 with the samples collected >14 days of symptoms onset with test agreement of 98.4% (kappa=0.963) (Table 1). The overall sensitivity of N-specific IgG ELISA was 94.9% (95% CI: 87.5%, 98.6%), which was better than S1-ELISA (Table 1) and, however, demonstrated slightly lower specificity than S1-ELISA with a kappa test agreement of 97% (kappa=0.937). The ROC for the overall assay was 0.97 (0.94, 0.99), with an overall PPV of 97.4% and NPV of 96.8% (Figure 2 and Table 1).

Comparative analysis between in-house ELISA

**Figure 3:**
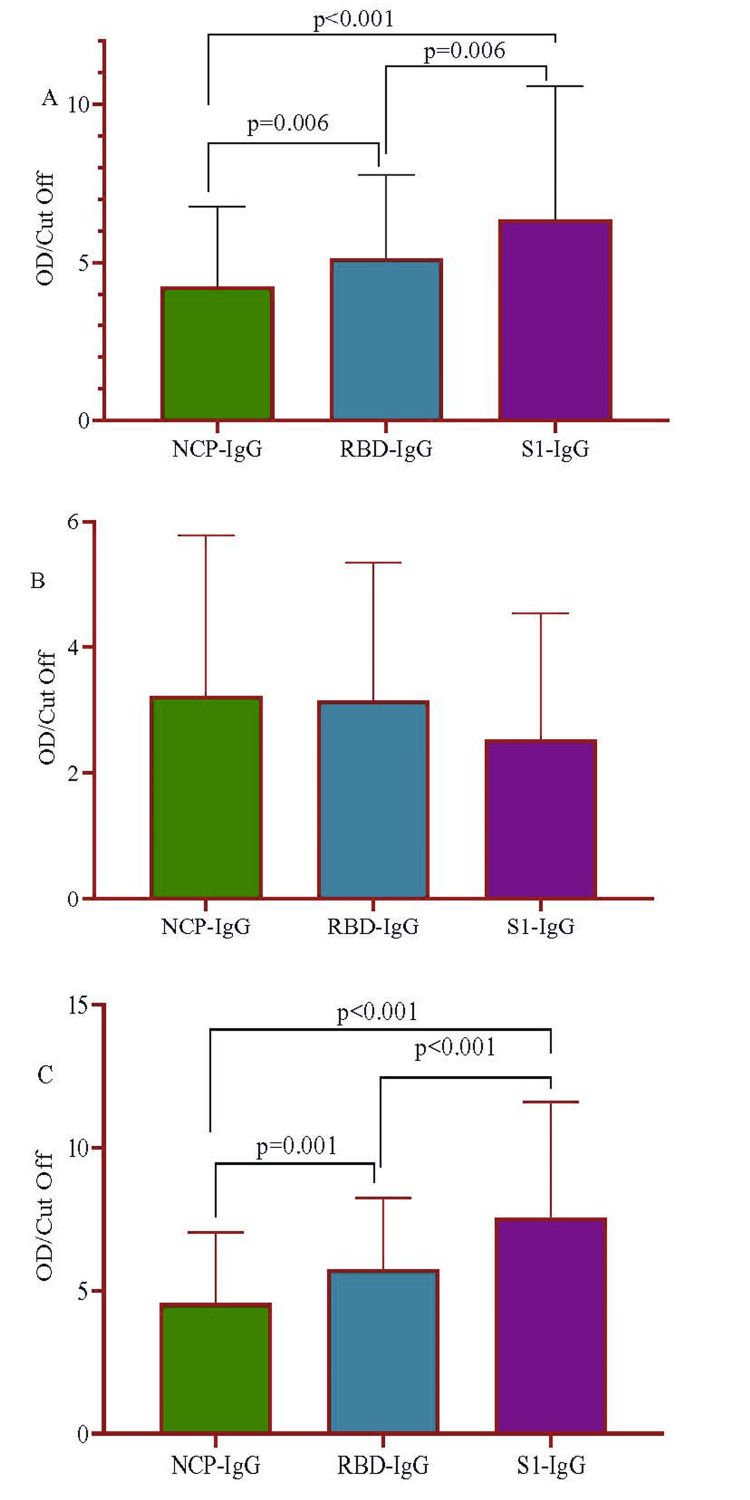
Detection of SARS-CoV-2 N-IgG, RBD-IgG, and S1-IgG among the SARS-CoV-2 confirmed patients with an overall period (A), <14 days (B), and >14 days (C). The ratio of OD/cut-off of N-IgG, RBD-IgG, and S1-IgG of the confirmed positives with SARS-CoV-2 was shown. Data are presented as mean with range (minimum and maximum). A non-parametric Wilcoxon signed-rank test was used to estimate the p-value. N: nucleocapsid; IgG: immunoglobin G; RBD: receptor binding domain; S1: spike-1; OD: optical density

After conducting a comparative analysis of the three in-house ELISA systems with identical panels, it was observed that the average optical density (OD)/cut-off value for S1 (6.34±4.24) was markedly greater than that of N (4.24±2.55; p<0.001) and RBD (5.12±2.66; p<0.001) (Figure 3A).

Interestingly, in <14 days of COVID-19 infection, S1 antibody titers (2.53±2.01) were lower than N (3.22±2.55) and RBD (3.15±2.19) (Figure 3B). However, the titer increased significantly at >14 days of the convalescent phase, i.e., S1 (7.55±4.04), N (4.56±2.48), and RBD (5.74±2.50) (Figure 3C), indicating S1 as an ideal seroprevalence marker.

Dengue sera cross-reaction with SARS-CoV-2 antigens

**Figure 4:**
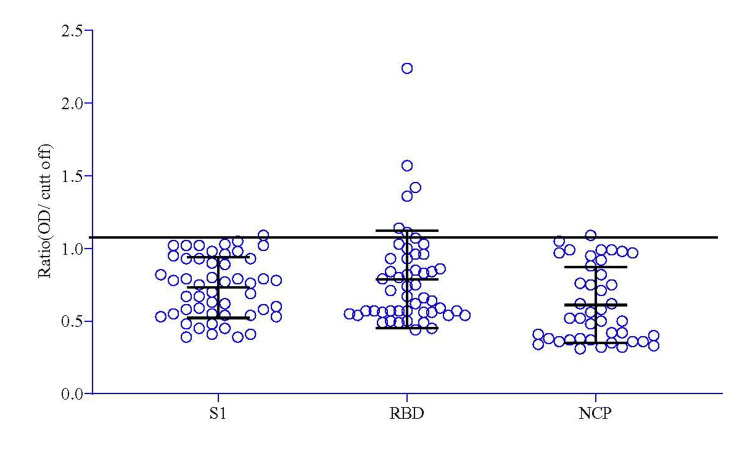
SARS-CoV-2 S1-IgG, RBD-IgG, and N-IgG were detected among the dengue-positive samples. The ratio of OD/cut-off S1-IgG, RBD-IgG, and NCP-IgG values of dengue-positives is shown. The reference line indicates the cut-off of the in-house ELISA methods. S1: spike-1; IgG: immunoglobin G; RBD: receptor binding domain; NCP: nucleocapsid protein; ELISA: enzyme-linked immunosorbent assay; OD: optical density

Fifty pre-pandemic dengue-positive samples (P2) and P1 were tested for cross-reaction against SARS-CoV-2 spike-1, receptor-binding domain, and nucleocapsid antigens (Figure 1). Interestingly, RBD reacted with six dengue sera; among those samples, one sample reacted with both N and S1 (Figure 4).

Spike-1 and RBD combination to improve sensitivity and specificity

To design an improved ELISA with minimal cross-reaction with dengue sera, we selected four pre-pandemic dengue sera (D 1-4), among which D2 and D3 cross-reacted with RBD, and D1 and D4 were non-reactive against S1 or RBD. We immobilized S1/RBD ratios of 100:0, 80:20, 60:40, and 0:100 onto the ELISA plate surface and tested the four dengue-positive samples in triplicate. We found minimal cross-reaction (OD/cut-off ratio) on 1.0:0.0 and 0.8:0.2 conditions with the dengue-positive samples (Table 2).

**Table 2:** OD/cut-off ratios of selected dengue samples on different combinations of S1 and RBD OD: optical density; S1: spike-1; RBD: receptor binding domain OD/cut-off >1.1 was considered a cross-reaction (in bold and italics)

Sample ID	S1: RBD	S1: RBD	S1: RBD	S1: RBD
	1.0:0.0	0.8:0.2	0.6:0.4	0.0:1.0
D1	0.791262	0.635246	0.673077	0.959538
D2	0.718447	0.823775	1.098266	1.569231
D3	0.708738	0.938525	1.202312	2.242308
D4	0.747573	0.588462	0.647541	0.867052

When 80:20 of S1: RBD was immobilized and P1, P2, P3, and P4 samples were challenged, the combination could successfully detect 84.2% (95% CI: 60.4%, 96.6%) of confirmed cases in P3 samples with 77.9% (kappa=0.819) test agreement (Table 3).

**Table 3:** Sensitivity, specificity, and ROC analysis for S1 and RBD combination (0.8:0.2) IgG in RT-qPCR positive against SARS-CoV-2 and negative controls S1: spike-1; RBD: receptor binding domain; CI: confidence interval; ROC: receiver operating characteristic; PPV: positive predictive value; NPV: negative predictive value; IgG: immunoglobulin G; RT-qPCR: reverse transcription-quantitative polymerase chain reaction Kohen's kappa test was used to estimate the test agreement.

Protein	Days	Sensitivity (95% CI)	Specificity (95% CI)	ROC (95% CI)	PPV (95% CI)	NPV (95% CI)	Kappa	Test agreement	Accuracy
Combination (S1: RBD =0.8:0.2)	≤ 14 (P3)	84.2(60.4, 96.6)	97.7(93.5, 99.5)	0.91(0.82, 1.0)	84.2(60.4, 96.6)	97.7(93.5, 99.5)	0.819	77.9%	92.0%
	> 14 (P4)	100(94.0, 100)		1.00(0.99, 1.0)	98.4(91.2, 100)	100(97.2, 100)	0.988	99.5%	99.5%
	Overall	96.2(89.3, 99.2)		0.97(0.95, 0.99	96.2(89.2, 99.2)	97.7(93.5, 99.5)	0.939	97.1%	98.0%

**Figure 5:**
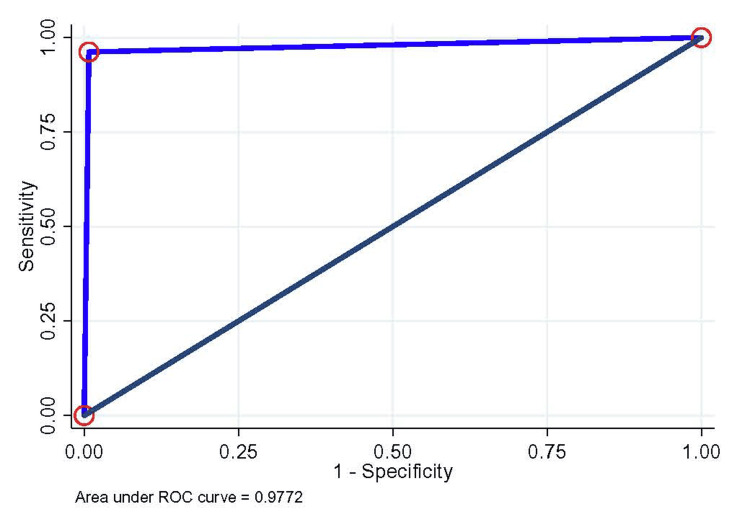
Detection of SARS-CoV-2 IgG against S1 and RBD combinations among the overall participants; the area under the ROC curve was considered both true and test cases S1: spike-1; IgG: immunoglobulin G; RBD: receptor binding domain; ROC: receiver operating characteristic

With a test agreement of 95% (kappa=0.988), the test kit was able to detect 100% (95% CI: 94.0%, 100%) of P4 samples. The dual antigen COVID-specific assay presented an overall strong test agreement of 97.1% (p<0.001) with a sensitivity and specificity of 96.2% (95% CI: 89.3%, 99.2%) and 97.7% (95% CI: 93.5%, 99.5%), respectively (Table 3). Similarly, the ROC, PPV, and NPV for S1: RBD ELISA were 0.97 (0.95, 0.99), 96.2%, and 97.7%, respectively (Table 3 and Figure 5).

## Discussion

The limitations of molecular diagnostics, specifically the detection of viral ribonucleic acid (RNA) in the later stages of COVID-19 diagnosis, make it necessary to implement serological tests. These tests help provide a better understanding of the disease's progression and enable the identification of asymptomatic infections [32]. Scientists worldwide are exploring the development of various immunoassays, leading to the availability of tests based on ELISA, lateral flow immunoassays (LFIA), chemiluminescent assays, flow-through dot-blot assays, and more [29]. However, the available serological assays have indicated lower sensitivities, particularly for LFIA, even when conducted more than 21 days after the onset of symptoms [33]. Developing immunoassays with higher sensitivity and specificity is essential to avoid false-positive and false-negative outcomes.

Viral entry into cells is facilitated by binding the RBD part of the S1 protein and human (part of S1) angiotensin-converting enzyme-2 (hACE-2). 445-456, 473-477, and 484-505 residues of the RBD part are responsible for the interaction, ultimately leading to a cascade of events [34,35]. Upon infection with the SARS-CoV-2 virus, the anti-RBD IgG thus produced lingers within the body for up to 75 days. These antibodies do not cross-react with other known circulating human coronaviruses [36,37]. Henceforth, the developed ELISA assay can detect and study seroconversion and vaccine efficacy [31].

This study aims to develop local ELISA assays targeting antibodies against a combination of spike S1 protein and RBD. When S1, RBD, and N were immobilized individually, surprisingly, S1 showed the lowest sensitivity (68.4%) among the three antigens when challenged with samples collected <14 days after symptom onset (Table 1). A similar observation was found by Brochot et al., where RBD and N showed better sensitivity than S1 in the earlier phases of infection [38]. Despite RBD being a part of S1, the immobilized proteins' equimolar concentration can explain the early sensitivity phenomenon, where RBD is the dominant immune-reactive site [39]. However, all three antigens were good seroconversion markers for convalescent sera characterization, reaching a sensitivity close to 100% (Table 1). However, RBD was found to cross-react more (6/50) with dengue sera than the rest of the two (1/50) (Figure 4), an observation previously reported by Nath et al. [40].

Comparing our in-house N-specific IgG ELISA and commercially available chemiluminescence immunoassay (Roche) with P3 samples, we obtained equal sensitivity for N-specific IgG ELISA and RBD-ELISA while being higher when compared with that of S1-ELISA (Table 1). However, in P4 samples, N-IgG ELISA misidentified ~2% of the positive samples as negative at a rate similar to previously reported (Table 1) [41]. The presentation of the early inception of IgG against N can be attributed to the 90% amino acid homology of SARS-CoV. Exposure to SARS-CoV or other human coronaviruses may cause cross-reaction while declining the specificity [42,43]. Our observation of a higher titer of S1 antibodies than RBD and N in COVID-19 patients (Figure 3) is in congruence with the dynamic and kinetic heterogeneity of the antibody titers reported previously [31,44]. A similar observation was also reported by studies performed with children with multisystem inflammatory syndrome and other diseases [27,45].

In the dengue-endemic region, especially in the lower and lower-middle-income countries, dengue is primarily diagnosed using serological assays. In the COVID-19 pandemic era, cross-reaction of dengue samples in COVID-19 serological assays or vice versa creates misdiagnosis and generates false epidemiological data [10-13]. Yan et al. have reported cases where COVID-19 patients' sera cross-reacted with dengue IgG/IgM assays [11]. Additionally, others have said that the dengue-infected patient's sera cross-reacted with SARS-CoV-2 antigens [10,46,47]. On the other hand, Spinicci et al. reported low chances of a cross-reaction between these two diseases [48]. The target antigens and their quality may contribute to the variable outcomes of the assay kits.

Nath et al., using computational modeling with DENV antibody crystal structures, predicted 19 antigenic cross-reaction sites in the SARS-CoV-2 RBD domain, among which seven fall in the ACE-2 binding region (438-506) [40]. Similar cross-reactions can be found in the Masyeni et al. study, where DENV-positive sera cross-reacted in COVID-19 serological assay kits with RBD as the target antigens [10]. Lustig et al., on the other hand, using an in silico model, predicted the major cross-reacting site of spike protein to be the HR2 domain [46]. The latter reported a 22% cross-reaction of DENV sera with the COVID-19 assay targeting S protein (EUROIMMUN ELISA). However, their claims contradict their in silico result, as the assay kit used the S1 domain, in which the HR2 domain is absent [49,50]. Moreover, dengue pre-pandemic infection sera reacted with RBD (3/37), whereas S1 showed less cross-reaction (1/47) when they checked the IgG antibodies. Because RBD is a part of the S1 domain, a higher molar concentration of RBD can justify such a cross-reaction when the equimolar amounts of these antigens are compared. Furthermore, a higher cross-reaction was observed in S1 (10/47) compared to RBD (1/37) when they carried out the assay for IgA antibodies, which can be further explained by the polymeric forms of IgA, which can recognize a more comprehensive range of antigenic sites [51].

Co-immobilization of antigens, such as N and RBD/S, is found to augment the sensitivity of the immunoassays [52,53]. We aimed to improve the overall sensitivity of the assay as well as reduce the cross-reaction. When combinations of S1 and RBD were challenged with dengue sera, minimal cross-reaction was observed at an S1:RBD ratio of 80:200 (Table 2). Interestingly, the early sensitivity improved at this ratio compared to S1-ELISA and increased from 68.4 to 84.2 (Tables 1 and 3). Similarly, the specificity was found to be 97.7%, which improved when compared to RBD (95.4%) but reduced slightly concerning S1 (99.2%) (Tables 1 and 3). This observation indicates the RBD domain of S as the dengue cross-reacting part, but again, it is an essential one for overall sensitivity. Despite RBD being a part of S1, a lower molar ratio reduces cross-reaction with dengue-positive sera. Still, careful tweaking of the co-immobilized balance of RBD and S1 can improve sensitivity with a minimum compromise of specificity.

The rigorous and spontaneous mutation of SARS-CoV-2 has created chaos worldwide, occurring in multiple infection waves with several deadly variants [54-56]. Worldwide, proper vaccine distribution and heterologous doses have become a critical concern under these circumstances [57-59]. Besides, serological testing is required to assess the efficacy of different vaccines and understand booster doses' necessity [60]. Therefore, it is essential to reduce the plausible cross-reaction of serological tests to critically investigate the requirement of a booster dose along with the minimization of misdiagnosis, accurate prognosis, and sero-surveillance in suspected confirmed hospitalized, recovered, and vaccinated patients [60, 61].

While our findings are noteworthy, as they reveal cross-reactivity between anti-dengue antibodies against the SARS-CoV-2 antigens, it's important to acknowledge certain limitations. Unfortunately, we could not evaluate cross-reactivity with SARS-CoV and other prevalent coronaviruses due to budgetary constraints and limited facilities. Additionally, the relatively small sample size in the P3 group adds to the limitations of this study. Nevertheless, the minimal observed cross-reactivity suggests that the developed ELISA assay can be effectively utilized in regions where dengue is endemic. Moreover, spectrum biases do not influence these assays, further enhancing their applicability and reliability.

## Conclusions

The S1 and N proteins demonstrated a better seroprevalence marker for the analysis of COVID-19. Although RBD was found to be slightly cross-reactive with anti-dengue antibodies, it is crucial for the early diagnosis of COVID-19 antibodies in serum. Therefore, co-immobilization of S1 and RBD with an appropriate and stable ratio is suggested to minimize the plausible cross-reaction.
